# Acceptability of risk-tailored cancer screening among Australian GPs: a qualitative study

**DOI:** 10.3399/BJGP.2023.0117

**Published:** 2024-02-20

**Authors:** Kate LA Dunlop, Amelia K Smit, Louise A Keogh, Ainsley J Newson, Nicole M Rankin, Anne E Cust

**Affiliations:** The Daffodil Centre, a joint venture with Cancer Council NSW and Melanoma Institute Australia, University of Sydney, Sydney.; The Daffodil Centre, a joint venture with Cancer Council NSW and Melanoma Institute Australia, University of Sydney, Sydney.; Centre for Health Equity, Melbourne School of Population and Global Health, University of Melbourne, Melbourne.; Faculty of Medicine and Health, Sydney School of Public Health, Sydney Health Ethics, University of Sydney, Sydney.; Evaluation and Implementation Science Unit, Melbourne School of Population and Global Health, Faculty of Medicine, Dentistry and Health Sciences, University of Melbourne, Melbourne.; The Daffodil Centre, a joint venture with Cancer Council NSW and Melanoma Institute Australia, University of Sydney, Sydney.

**Keywords:** cancer screening, general practice, implementation science, risk assessment

## Abstract

**Background:**

Cancer screening that is tailored to individual risk has the potential to improve health outcomes and reduce screening-related harms, if implemented well. However, successful implementation depends on acceptability, particularly as this approach will require GPs to change their practice.

**Aim:**

To explore Australian GPs’ views about the acceptability of risk-tailored screening across cancer types and to identify barriers to and facilitators of implementation.

**Design and setting:**

A qualitative study using semi-structured interviews with Australian GPs.

**Method:**

Interviews were carried out with GPs and audio-recorded and transcribed. Data were first analysed inductively then deductively using an implementation framework.

**Results:**

Participants (*n* = 20) found risk-tailored screening to be acceptable in principle, recognising potential benefits in offering enhanced screening to those at highest risk. However, they had significant concerns that changes in screening advice could potentially cause confusion. They also reported that a reduced screening frequency or exclusion from a screening programme for those deemed low risk may not initially be acceptable, especially for common cancers with minimally invasive screening. Other reservations about implementing risk-tailored screening in general practice included a lack of high-quality evidence of benefit, fear of missing the signs or symptoms of a patient’s cancer, and inadequate time with patients. While no single preferred approach to professional education was identified, education around communicating screening results and risk stratification was considered important.

**Conclusion:**

GPs may not currently be convinced of the net benefits of risk-tailored screening. Development of accessible evidence-based guidelines, professional education, risk calculators, and targeted public messages will increase its feasibility in general practice.

## Introduction

Interest in risk-tailored population-based screening for cancer is rapidly increasing, with international trials exploring implementation of this approach.^1^^–^^4^ Risk-tailored screening aims to provide personalised cancer screening for the individual rather than the ‘one-size-fits-all’ population programmes designed for groups with defined characteristics. The tailoring of screening frequency, age range, and modality according to personal risk has the potential to improve health outcomes for those at high risk of cancer, while reducing screening programme costs and harms related to overdiagnosis and false-positive results for those at low risk.^5^^,^^6^ In Australia, publicly funded national programmes provide population-based screening for breast, bowel, and cervical cancer, while opportunistic screening, that is, either by patient request or incidentally by a doctor, is available for melanoma^7^ and prostate cancer.^8^ Risk-tailored screening for lung cancer is currently under review by the Australian Government following a recommendation to implement a national programme.^9^ GPs provide advice to patients on cancer screening and deliver testing for melanoma, and for cervical and prostate cancers.

Risk-tailored screening will require changes in clinical practice.^5^^,^^10^^,^^11^ For example, assessment of personal risk will likely be based on many different factors such as lifestyle behaviours, environmental risk factors, and personal genomic risk information, in addition to traditional risk factors of age, family history, and ethnicity. Screening frequency may need to be reduced (or perhaps not offered at all) for low-risk individuals. These potentially significant changes in practice to cancer screening and early detection programmes, and additional communication needs, present challenges for implementation.^12^ Thus, before implementation of risk-tailored screening, determining its acceptability to key stakeholders, including GPs, will be important for successful integration. Acceptability refers to the perception that a change in practice is agreeable^13^^,^^14^ and in the target population’s interest.^15^^,^^16^ Acceptability increases the likelihood that GPs will adhere to guidelines, although complexities related to sustaining adherence are well recognised.^17^ GP support for patients taking a test increases appropriate participation in screening.^18^

**Table:** How this fits in

Risk-tailored cancer screening has the potential to increase the benefits and reduce the harms of screening, with implementation foreseeable in the future. This approach will require a change of practice, particularly for GPs, therefore successful implementation will depend on its acceptability. This qualitative interview study found that GPs felt that risk-tailored screening was acceptable in principle; however, they had some concerns for their patients and practice. Strategies to reduce the potential for patient confusion and to support GPs to deliver risk-tailored screening will be needed to increase feasibility.

Very little is known about health professionals’ views on risk-tailored screening for cancer types other than for breast cancer.^19^ In the context of breast cancer screening, it is generally viewed as acceptable but some health professionals report concerns associated with reducing frequency or excluding screening altogether for those deemed at low risk.^2^^,^^20^^,^^21^ While implementation of risk-tailored cancer screening is foreseeable in the future,^5^^,^^7^^,^^22^ to the authors’ knowledge there is no study focusing specifically on GPs’ perspectives across cancer types. This study aims to explore Australian GPs’ views on the acceptability of risk-tailored screening across cancer types and to identify barriers to and facilitators of implementation. Exploring multiple cancer types in one study enables focus on acceptability of the concept of risk-tailored screening and the consequent change of practice that underpins implementation of this new approach.

## Method

### Recruitment and participants

Semi-structured interviews were conducted with GPs across Australia. Most of the GP participants were recruited through patients who, after participating in a cancer prevention study,^23^ provided written consent for their GP to be contacted by the study researchers. Two GPs were recruited through professional networks to ensure that a diversity of years of experience in general practice was represented.^24^ GPs were mailed an invitation letter with a participant information sheet, consent form, and reply-paid envelope, and were followed up with a phone call 2 weeks later if no response had been received. GPs were not asked to discuss their patient and no patient health information was recorded.

### Data collection

The interview guide (see Supplementary Box S1 for details) was informed by Proctor *et al*’s^13^ outcomes for implementation research framework to ensure questions explored all key aspects of implementation, with a particular focus on the concept of acceptability, that is, the perception among implementation stakeholders that a given treatment, service, practice, or innovation is agreeable, palatable, or satisfactory. During the interview, participants were asked what they thought about the concept of risk-tailored screening generally, in relation to different cancer types, and barriers to and facilitators of integration into general practice. Interviews were conducted between April 2019 and May 2021 by the first author via telephone and audio-recorded. Interviews were suspended between February and June 2020 because of COVID-19-related impacts on GP workload. GPs were reimbursed for their time with an AUD $80 gift voucher.

### Data analysis

Interviews were professionally transcribed and de-identified. Data were analysed in two separate rounds. First, inductive thematic analysis guided by Braun and Clarke’s six-step process^24^ was conducted to explore and understand GPs’ experiences and perspectives. All research team members initially read three transcripts to familiarise themselves with the data and met to discuss and mark sections of the transcripts relevant to the research questions. Next, transcripts were independently read by two researchers who compared the data within and across interviews to identify common patterns and develop initial codes. The first author searched the transcripts again, reviewing codes to look for broader patterns of meaning and generate preliminary themes. Feedback on coherence of preliminary themes presented with definitions and exemplar quotes was sought from the research team and the coding framework was refined. Reporting of findings was informed by Standards for Reporting Qualitative Research recommendations.^25^

During the second round of analysis, transcripts were re-read by the first author and, along with existing themes and subthemes, were coded using a deductive approach into the Tailored Implementation for Chronic Diseases (TICD) checklist.^26^ The TICD checklist is a comprehensive, integrated checklist of 57 determinants of practice grouped in seven domains for the implementation of new health interventions. It was selected as a framework to guide identification of barriers and facilitators relevant to implementation. Determinants of practice are factors that might prevent or enable healthcare improvements, and coding barriers and facilitators according to this established framework allowed researchers to determine which specific determinants were present in the data. Findings were discussed and agreed with the wider research team, including identifying the most relevant TICD determinants of practice, and barriers and facilitators.

## Results

A total of 20 GPs were interviewed. Table 1 provides characteristics of GP participants and their experience. A total of 101 GPs were invited to participate and 18 (18%) provided consent, with an additional two GPs recruited via professional networks, one with limited experience (practising as a GP for 12 months) and one with extensive experience (>30 years). Interviews were on average 23 minutes in length (range 10–41 minutes).

**Table 1.** Characteristics of GP participants (*N* = 20) and their experience

**Characteristic**	***n* (%)**
**General practice type**	
Medical practice	19 (95)
Skin cancer clinic	1 (5)

**Years’ experience in general practice**	
1–9	7 (35)
10–19	2 (10)
20–29	4 (20)
≥30	7 (35)

**Gender**	
Female	10 (50)
Male	10 (50)

**Australian State/Territory**	
New South Wales	12 (60)
Queensland	2 (10)
Victoria	2 (10)
Western Australia	2 (10)
South Australia	1 (5)
Australian Capital Territory	1 (5)

Five main themes were identified by the inductive thematic analysis and the coding framework, with corresponding subthemes presented in Box 1.

**Box 1.** Coding framework (inductive thematic analysis) related to acceptability of risk- tailored cancer screening (subthemes are given below each main theme)

**Evidence of benefits for risk-tailored screening** Benefits Harms Evidence **Concern for the patient** Confusion/conflicting messages Patient responses **Reservations related to own practice** Impact on everyday practice Issues related to genomic risk information Fears **Some cancers are more suited than others** Cancer type Screening test **Engaging and enabling risk-tailored screening by GPs** Education needs GP interest Accessing evidence

### Evidence of benefits for risk-tailored screening

Participants generally reported that a risk-tailored approach to screening would have benefits, particularly in terms of reducing needless screening and potential screening-related harms, as well as increasing motivation for patients at high risk to attend screening. Many participants felt they would be more likely to adopt a risk-tailored approach if the evidence was convincing of the net benefits, valuing patient needs over cost–benefit to the broader health system. Risk-stratifying patients based on lifestyle and family history was not, however, seen as a new concept but rather something GPs do in routine practice. Participants felt that including additional risk information into risk stratification was potentially adding value to routine practice:

*‘That’s* [incorporating a range of risk factors] *what we instinctively do anyway.’*

(Participant [P]16, >30 years’ experience)

Participants acknowledged potential benefits of a risk-tailored approach, with some referring to reducing the chance of false-positive screening results and thus avoiding unnecessary anxiety, often referring to examples in their own practice:

*‘I’ve come across in general practice a lot of anxiety and stress that’s come from, I guess, false positives on screening of otherwise a low-risk person in terms of investigation of breast lumps or … in terms of men having prostate investigations as well. So, I think it’s definitely something moving forward and a more individual, personalised approach could be a good change.’*

(P11, 1–9 years’ experience)

Many participants acknowledged a potential cost–benefit recognising the merit in allocating resources to those who would benefit most:

*‘So, probably from an economic point of view, if you could then fund the screening processes that are going to be more likely to pick something up, then I guess that’s a bit more cost effective*.*’*

(P12, >30 years’ experience)

However, for some, it was unclear whether the benefits of risk-tailored screening would outweigh harms. Participants expressed a level of uncertainty about integrating a new approach without high-quality evidence and clear guidelines to support a change:

*‘How clear are the percentages going to be to move someone into extra screening, I suppose, as opposed to someone who is just across the board?’*

(P05, >30 years’ experience)

Another further explained the importance of strong evidence:

*‘If the science is backing it, I think that’s fair. If it’s more about cost and convenience … that would be very unfair.’*

(P09, 20–29 years’ experience)

### Concern for the patient

Participants spoke at some length about their concern for how patients may respond to risk information and screening advice. Many GPs shared the view there was potential for confusion associated with new advice that may be seen to conflict with previous screening recommendations. Participants placed high value on maintaining patient trust in their role as primary care providers, some explaining their preference to prioritise individual patient needs over population needs. Concern about the potential to increase patient anxiety for those deemed at high risk was also reported by a few participants, questioning the benefit to the patient of knowing personal genomic risk information:

*‘I also wonder whether if it will make patients a whole lot more worried. I think if a patient was to have genetic screening done at the age of forty and they were to find out that their risk of getting melanoma by the age of fifty was ten times higher than the population, I do wonder.’*

(P10, 1–9 years’ experience)

Participants frequently referred to how patients respond differently to screening advice according to their experience, highlighting the complexity of the patient consultation and predicting outcome. One participant explained how a patient’s current screening practice may impact on their response to reduce or stop screening altogether:

*‘I think they’d probably accept it because it would be less bother for them — they’d appreciate not having to go and get tested. If they’re already used to it, then they’d probably want to continue it, even if they found out that they weren’t* [at high risk]*.’*

(P07, 20–29 years’ experience)

Overwhelmingly, participants were concerned with how patients would respond to potentially confusing public messages and the impact this may have not only on patient response to screening and but also on the GP’s relationship with patients:

*‘I think the patients would be thinking that we are being rather slack actually and that we are not looking after the interests of the patients, given we promote it so much, that screening is so important, and then we would be backing away from it all.’*

(P09, 20–29 years’ experience)

One GP explained the challenges of following guidelines when to do so would conflict with their goal of maintaining patient trust:

*‘GPs are pragmatists, I suppose, so even though I try and follow evidence, but sometimes you do things that aren’t totally evidence-based but are more to try and maintain a therapeutic relationship with your patient and trust.’*

(P15, 1–9 years’ experience)

### Reservations related to own practice

While participants were cognisant of the potential benefits of risk-tailored screening, many expressed reservations related to changing current practice. Some reported that ongoing time constraints on patient consultations would mean they were less likely to adopt a new approach that was seemingly more complex, although introducing personal genomic risk information as a risk factor was not seen as a barrier. Some felt that routine screening appointments for all patients had value in early detection that may be lost in a risk-tailored screening approach. In particular, participants were worried about missing a cancer diagnosis in those at low risk:

*‘But the disadvantage is if it wasn’t the whole population who was able to access it, then some people might fall through the net, which is what a screening process is usually for, to get everyone at risk, not just a specific population.’*

(P07, 20–29 years’ experience)

Two participants expressed reluctance to forgo routine screening for all patients, considering it a missed opportunity for regular patient care and managing challenges in today’s climate of consumer self-management of health:

*‘If you get people in regularly, they ask you things. It’s very difficult if they just look after their own health and buy things online … So, the simple fact of getting them to a doctor is often more valuable, regardless of what they came for.’*

(P16, >30 years’ experience)

Some participants indicated that risk-tailored screening may not be a priority at this stage when the current practice seems to be working and that limited time for patient care was an ongoing challenge:

*‘The limit though is the time constraints in general practice, I think it’s the biggest barrier.’*

(P10, 1–9 years’ experience)

While a few were unsure about whether to include personal genomic risk information as part of the personal risk assessment, many participants felt comfortable to discuss genetic information and potential implications with patients following education:

*‘I think if we were provided with the right training on what it means in terms of potential negative outcomes, such as potential effects on, say they want to apply for life insurance, what effect it could have on that, or the emotional toll it can have on the patient, how to deal with that, how confidential the genetic information is, I think I would be comfortable to do that. But if I wasn’t well trained, I’d feel uncomfortable.’*

(P06, 1–9 years’ experience)

### Some cancers are more suited than others

When asked whether cancer type would make a difference to acceptability of risk-tailored screening, participants identified cancer frequency in the population and the type of screening test as factors that may have an impact. They felt that patients would be more amenable to reducing screening frequency if the test was invasive. There was general consensus that if a cancer was common and patients were already engaged in screening, reducing screening frequency might not seem to be in the patient’s best interest and be less acceptable to participants and to their patients:

*‘I think if the cancer is a common cancer (e.g. breast cancer) and the cancer is more likely to cause more negative outcomes, then I think that unfair feeling (for low-risk patients) might be heightened, compared to a cancer that’s much less common, for example, ovarian cancer.’*

(P06, 1–9 years’ experience)

Participants mostly associated patient acceptability of reduced screening frequency with invasive screening tests. Participants identified patients’ acceptance, despite initial hesitancy, of the recent decrease in frequency and increase in starting age for cervical cancer screening in Australia^27^ to be related to the invasive nature of testing. Some described encounters with patients who requested screening only for less invasive tests, as one GP explained:

*‘Men are quite happy to have a blood test to look for their PSA* [prostate-specific antigen] *level, but if you set a screening test with a digital rectal examination, many men would decline that as a screening test.’*

(P10, 1–9 years’ experience)

This GP went further to say that the unpleasantness as well as the invasiveness of a screening test may also impact on acceptability:

*‘While a mammogram is probably a bit uncomfortable, people are quite happy to have a mammogram done. Whereas many people don’t like doing bowel cancer screening because it involves collecting a faeces sample, which people don’t like doing.’*

(P10, 1–9 years’ experience)

The type of test was also relevant to how some participants felt about screening frequency. One reported that they would more likely continue screening for melanoma because of the simplicity of skin checks. Many participants also expressed fear of missing a diagnosis for melanoma above other cancer types, describing the need to continue skin checks and include strategies to avoid missing an opportunity for early detection:

*‘I think obviously melanoma is very tricky. As a general practitioner, you always hope you don’t miss something like a melanoma. So not screening is something that would concern me.’*

(P15, 1–9 years’ experience)

### Engaging and enabling risk-tailored screening by GPs

Many participants identified that engaging GPs in education to support a change in practice would be a challenge considering their competing priorities and time constraints. For some, this was the biggest barrier for implementing this new approach. However, GPs did associate guidelines and point-of-care tools as a way to integrate a new approach and support more complex consultations. The perceived need for GP education varied, with some valuing a comprehensive overview of risk-tailored screening. However, others reported education was not a solution, though often offered, to the ongoing time constraints of general practice. Most did request training associated with communicating risk assessment results:

*‘I think* [we need] *education about how GPs can interpret the test results and then develop that language to be able to communicate those test results into information that the patient can understand.’*

(P10, 1–9 years’ experience)

Guidelines were almost unanimously identified as essential to integrating any new approach into practice. Participants explained that guidelines not only meet their information needs but also provide a source of evidence to support often challenging decision making in practice. However, equally important was being able to access them at point of care:

*‘I find guidelines very, very helpful, because you have to treat patients and it’s a risk, so there’s always a risk of missing things and if you follow the guidelines, at least it’s something you can hold on to … There are lots of guidelines, but they’re all scattered all over the place and that makes it more difficult to access them when you need them.’*

(P03, 1–9 years’ experience)

GPs discussed positive experiences with risk calculators, suggesting they provide a practical and helpful way forward:

*‘Certainly having calculators built into practice software is very useful, things like the cardiovascular risk assessment, which is very easy to plug in people’s smoking status and diabetes and come out with a number and a graph that you can explain to patients. Whether there was something similar to use to explain risk stratification, that would be handy.’*

(P10, 1–9 years’ experience)

Additional quotes are included in Box 2 to support themes.

**Box 2.** Additional exemplar quotes to support themes related to acceptability of risk-tailored cancer screening

**Evidence of benefits for risk-tailored screening** Benefits: *‘Tailoring would be good because it might mean that you’d focus on those higher-risk people and it might improve the pick-up rate for different conditions.’* (P15, 1–9 years’ experience) Evidence: *‘Look, I would happily follow guidelines. There just has to be enough sense behind them for me to feel comfortable arguing the case with the patient to say, “ Well we’ve been pursuing this as dogma, now we’re changing to this”, so I’d have to be comfortable with the science behind it.’* (P09, 20–29 years’ experience) **Concern for the patient** Confusing messages: *‘It’s all these mixed messages let alone for general practice, but for the public. So it* [public health messaging] *has to be carefully thought of rather than alarming the public about a change that can be confusing.’* (P01, 1–9 years’ experience) Patient responses: *‘Look, I can see that it would have its benefits, but I could see that it could have some negative impacts as well in terms of potentially increasing people’s anxiety about their risk.’* (P12, >30 years’ experience) **Reservations related to own practice** Impact on everyday practice: *‘I think the barriers are health system barriers, that is a time-based system, the lack of these sort of tools that are freely available in the workspace of general practitioners.’* (P01, 1–9 years’ experience) Issues related to genomic risk information: *‘Obviously* [personal genomic risk information] *is useful in theory. In practice, how do we ask those questions and then input them into some sort of algorithm in a timely manner?’* (P14, 1–9 years’ experience) Fears: *‘I have to admit that skin cancer is something that terrifies me. It’s one of my areas of weakness and I do end up actually referring a lot of my patients to a skin clinic for assessment these days so that I don’t miss anything.’* (P17, 10–19 years’ experience) **Some cancers are more suited than others** Cancer type: *‘I think especially breast cancer seems to fear lots and lots of women, so reducing screening or not screening at all I can see that would upset a lot of people.’* (P03, 1–9 years’ experience) Screening test: *‘I think perhaps because a colonoscopy is a lot more invasive than having a mammogram, people might be a little bit happier about saying, “ Oh great, I don’t have to have that again or I don’t have to have it every five years, every ten years.”’* (P09, 20–29 years’ experience) **Engaging and enabling risk-tailored screening by GPs** Education needs: *‘I think we do need better education around risk stratification, but not only about identifying high risk, but also what to do about those at low risk, so there’s a two-pronged educational aspect of it.’* (P01, 1–9 years’ experience) GP interest: *‘The biggest barrier would be just access to educating GPs about the change and the different data and why there may be a benefit.’* (P11, 1–9 years’ experience) Accessing evidence: *‘If you have the tools and education about a particular thing, it’s got to be a central area where the GPs have to access it. It’s got to be that resources are available for all those things so that if we have a patient in front of us that you’re worried about, then we can then say, “Okay, what’s the current thinking about it, what’s the current treatment? ”’* (P20, >30 years’ experience)

### Barriers to and facilitators of implementation

During the second round of analysis, themes identified and discussed above were then deductively coded into six relevant TICD domains (guideline factors; individual health professional factors; patient factors; incentives and resources; capacity for organisational change; and social, political and legal factors), with barriers and facilitators highlighted for each. One TICD domain, ‘professional interactions’, and some determinants of practice were not relevant or infrequently discussed and these were omitted from the reporting. See Supplementary Table S1 for details of relevant TICD domains and determinants, with definitions included.

Box 3 presents barriers to and facilitators of implementation reported in the data mapped to relevant determinants of practice of the TICD checklist. This provides a means to focus on the factors that impact on implementation specifically for risk-tailored screening. While facilitators are mapped to most determinants of practice, GPs did not report facilitators related to the determinant ‘priority of necessary change’ in the TICD domain ‘capacity for organisational change’, suggesting that GPs may not feel they have the need or means to enable change.

**Box 3.** Barriers and facilitators identified in the data, linked to the TICD checklist

**TICD domain headings with relevant determinants of practice**	**Barriers to implementation identified in the data mapped on to the TICD checklist**	**Facilitators of implementation identified in the data mapped on to the TICD checklist**
**Guideline factors**		
Quality of evidence Strength of recommendation Accessibility of recommendation Feasibility	Lack of certainty that benefits outweigh harms Perceived lack of easy access to resources at point of care	Accessible evidence-based guidelines

**Individual health professional factors**		
Skills needed to adhere Agreement towards guidelines Emotions Nature of the behaviour/capacity to plan to change	Lack of expertise communicating risk assessment results Lack of consensus on approach to education Reluctance to reduce screening frequency for common cancers/potential fidelity Fear of missing a cancer Lack of time for risk-tailored patient discussions	Professional education: communicating risk assessment results GP expertise with screening programmes/risk factors Perceived benefit of reduced screening frequency where screening tests are invasive

**Patient factors**		
Patient needs Patient beliefs and knowledge Patient behaviour	Patient resistance to screening advice Concern about patient anxiety related to high-risk results Patient response/confusion from changes in screening advice	Professional education — communicating risk assessment results Targeted public messages

**Incentives and resources**		
Continuing education system Assistance for clinicians	Lack of time for education/to become an evidence-based provider	Evidence-based guidelines and risk calculators

**Capacity for organisational change**		
Priority of necessary change	Lack of perceived priority to change practice	

**Social, political and legal factors**		
Economic constraints on the health budget	Reluctance for change if cost-effectiveness is the only reason for change	Potentially cost-effective

*TICD = Tailored Implementation for Chronic Diseases.*

## Discussion

### Summary

This study aimed to present a detailed analysis of the views of Australian GPs on risk-tailored screening across different cancer types and to identify relevant barriers to and facilitators of implementation. Participating GPs found risk-tailored screening to be acceptable in principle, recognising potential benefits in targeting those at high risk of cancer. However, the findings also highlighted that GPs had significant concerns about a possible change in screening advice that could potentially cause confusion. Reducing screening frequency or excluding those deemed at low risk from a screening programme may not be acceptable, especially for common cancers and for those already engaged in screening. However, some GPs thought a reduction of screening tests that are invasive, such as digital rectal examination for prostate cancer, may be more acceptable. Reservations about risk-tailored screening included fear of missed cancer diagnosis, particularly for melanoma, lack of certainty that benefits outweigh harms, loss of benefits of routine screening appointments for all patients, and time constraints.

With regards to barriers and facilitators, the inclusion of additional risk factors for risk assessment, in particular lifestyle and personal genomic risk information, was not considered a barrier. This is an important finding for implementation, although GPs reported the need for education in communicating a more complex risk assessment. A perceived lack of easy access to resources at point of care and a lack of a clear approach to education were challenges. This study indicates that currently GPs may not be convinced of the net benefits of risk-tailored screening or that a change of practice is a priority. However, GPs’ expertise in screening programmes and acknowledgement that a risk-tailored screening approach is foreseeable in the future suggest that engaging GPs early is important. Accessible evidence-based guidelines, professional education, risk calculators, and targeted public messages were identified as useful facilitators to support practice, as were the integration of risk-tailored approaches into general practice workflows that are not disruptive or burdensome.

### Strengths and limitations

The study sample was relatively small but provided detailed insights from a range of practising GPs. The two-step approach to analysis enabled the capture of the GP perspective (thematically) and the identification of relevant barriers and facilitators (informed by an implementation framework). The COVID-19 pandemic was a limitation that extended the interview period and impacted on GP recruitment. The increased workload related to COVID-19 limited availability and accessibility of GPs in some practices, potentially affecting the representativeness of the sample. However, equal numbers of males and females were recruited, and the sample represented a range of years of experience in general practice.

### Comparison with existing literature

Risk-tailored screening has generally been found to be acceptable by health professionals for breast cancer.^20^^,^^28^^,^^29^ Recent studies that specifically include primary care practitioners’ views report uncertainty about risk-tailored screening related to reliability of risk estimate accuracy^2^ and evidence of benefit for low-risk patients^21^^,^^30^ consistent with the findings of the current study. Interventions that demonstrate clear and consistent clinical evidence of benefit have been shown to facilitate implementation in primary care.^31^ Quality of evidence was a key determinant of practice in the current study, and establishing accessible clinical practice evidence-based guidelines before implementation, recognised also by others,^29^^,^^30^ will be crucial.

A framework-informed approach was used in this study to identify barriers and facilitators that warrant further investigation. Implementation determinant frameworks have previously guided identification of opportunities to better engage general practice in a colorectal cancer screening quality improvement intervention;^31^ and to better understand the complexity of adherence by a range of clinicians to recommendations in melanoma guidelines.^32^ Key determinants of practice identified using the TICD framework in the current study were ‘patient beliefs and knowledge’ and ‘patient behaviour’ in response to screening advice. Other studies^2^ similarly report the potential for patient confusion related to public messaging and the importance of focusing on clear public communication as a facilitator, strengthening this recommendation by GPs in the current study. Being stratified into a high-risk group was not previously seen as having a negative impact on patients’ emotional wellbeing,^33^ although GPs in the current study expressed concern about increasing anxiety for patients deemed at high risk of cancer. Guidance for health professionals on risk communication is a recurring theme in the literature,^29^ including providing personal genomic risk information, and represents a key facilitator. The current study found mixed responses to the requirement for education and type of education.

Consumer perspectives on the acceptability of risk-tailored cancer screening has been reported by others, including reluctance to accept a reduction in breast cancer screening frequency for women at low risk in Australia^34^ and internationally.^35^^–^^37^ Koitsalu *et al*^15^ reported that a reduction in screening frequency appeared to raise more opposition and hesitance among women for breast cancer than among men for prostate cancer. Consistent with GP views in the current study, risk-tailored screening has been seen as more acceptable by some consumers for cancers with invasive screening tests, such as for bowel cancer;^38^ however, this was in reference to colonoscopy — a screening intervention that in Australia is recommended only for people at increased risk of bowel cancer. Risk-tailored screening for melanoma was found to be acceptable by consumers, although those who reported already receiving regular skin checks expressed reluctance to forgo screening if at low risk.^38^ However, in the current study GP participants were more concerned, expressing fear of missing a cancer diagnosis especially for melanoma. This may be particularly relevant to the Australian context because of its high melanoma rates.^39^

Consistent with the findings of this study, the financially and time-constrained setting of primary care has been identified as a barrier.^29^^,^^30^ While the current study focused only on GP perspectives, reorganising human resources to optimise the workforce,^11^ involving other primary healthcare professionals in delivery,^40^^–^^42^ may be helpful for successful implementation.

We can look to human papillomavirus (HPV) screening for cervical cancer to learn some lessons on communicating about a reduction in screening frequency and the trade-off between benefits and harms of cancer screening at different ages or risk levels. In 2017, Australia’s National Cervical Screening Program made the transition from cytology-based screening from age 18 years to HPV-based screening from 25 years. Concerns among women and healthcare providers were reported, especially about the longer interval and later starting age for screening, and resulted in additional unexpected demand for colposcopy.^43^ While the National Cervical Screening Program does not require a preliminary risk assessment of personal genomic or lifestyle risk information in line with the approach discussed in the current study, follow up and testing is tailored based on risk-based clinical guidelines. Lessons learnt from this programme renewal are relevant for implementation of risk-tailored screening for other cancers, and include that concerns may have been reduced through earlier and more extensively delivered information to healthcare providers, reach and credibility of communication about the programme changes may have been improved with better coordination of stakeholder support between government and non-government organisations, and, where there is uncertainty about appropriate clinical management or testing, providers need training about the rationale for updated guidelines.^43^

GPs are central to discussions about cancer prevention and screening with patients, yet cancer screening programmes usually operate in a siloed manner. Australia has introduced a National Cancer Screening Register^44^ that provides digital access, including integrated clinical software, to screening information and management of participation in bowel and cervical screening programmes. This resource has the potential to meet many of the needs mentioned by GPs in this study, including accessible guidelines for multiple cancers in one place and a platform for risk calculators. However, it was not raised by participants, and few may be aware of its existence. This type of register may be one way of making screening easier for GPs by offering a one-stop shop for screening. Screening for multiple cancers at one time has the potential to reduce morbidity and mortality, as well as offer substantial cost savings.^45^

### Implications for research and practice

This study contributes novel findings about GPs’ views of risk-tailored cancer screening, which adds to the knowledge base before possible implementation in the Australian population. GPs find risk-tailored screening acceptable but have concerns for patients and the public related to a change in screening advice. Development of accessible evidence-based guidelines, professional education, and risk calculators will increase its feasibility in general practice. The assessment of personal cancer risk in a risk-tailored cancer screening approach may provide a mechanism to initially align the operation of screening programmes and is a potential area for research. Further research in primary care should focus on the optimal way to communicate the balance of benefits and harms of risk-tailored cancer screening to patients, and on pathways or options for those at low risk. 

## Data

Reasonable requests for data may be sent to anne.cust@sydney.edu.au (project reference: 2018/941).
